# Behaviors and influencing factors of Chinese oncology nurses towards hospice care: a cross-sectional study based on social cognitive theory in 2022

**DOI:** 10.1186/s12904-024-01385-8

**Published:** 2024-02-23

**Authors:** Jing Zhao, Yu Wang, Binbin Xiao, Fucheng Ye, Jianfei Chen, Yingjuan Huang, Ting Li, Xiaoli Chen, Hongmei Ma, Qing Zhang, Zhijie Zou

**Affiliations:** 1 School of Nursing, Wuhan University, Located on No. 115 Donghu Road, Wuhan, Hubei province 430071 China; 2 Renmin Hospital of Wuhan University, No.238 Jiefang Road, WuChang Distirct, Wuhan, Hubei Province 430060 China; 3 Wuhan University of Technology, Wuhan, China; 4 Hubei Cancer Hospital, Wuhan, China

**Keywords:** Hospice care, Nurse, Behavior, Oncology, Social cognitive theory

## Abstract

**Background:**

Although there is growing demand for hospice care in China due to its aging population and increasing cancer rates, the sector remains slow to expand. Oncology nurses are the primary providers of hospice care, but little is known about their behaviors towards hospice care and related factors.

**Methods:**

This cross-sectional study conveniently sampled 933 oncology nurses from six grade A tertiary hospitals in Hubei Province between January to March 2022. The questionnaire was composed of seven parts: general information (including sociodemographic and work-related information), hospice care behaviors, hospice care knowledge, hospice care attitudes, hospice care self-efficacy, hospice care outcome expectancy, and hospice care environment. Data were analyzed using descriptive analysis, independent sample *t*-tests, one-way ANOVA, Pearson’s correlation, multiple linear regression, random forest regression, and BP neural network model analysis.

**Results:**

A total of 852 questionnaires were valid. The mean score of hospice care behaviors was 50.47 ± 10.56, with a mean item score of 3.61 ± 0.75. The three highest scoring behaviors were “pain assessment of patients (4.21 ± 0.91)”, “satisfying the physical and mental needs of dying patients (4.04 ± 0.92)”, and “creating good relationships between the medical staff and family members (4.02 ± 0.87)”. The two lowest-scoring behaviors were “proactively recommending medical institutions for hospice care to terminally ill patients and their families (2.55 ± 1.10)” and “proactively talking to patients and families about death-related topics for patients who are critically ill and cannot be reversed (2.87 ± 1.03).” Multiple linear regression, random forest regression, and BP neural network models all showed that the frequency of sharing hospice care experiences with colleagues, hospice care attitudes, hospice care self-efficacy, and hospice care environments were positively associated with hospice care behaviors.

**Conclusions:**

The frequency of hospice care behaviors among Chinese oncology nurses is generally at a moderate to high level. The results provide a basis for promoting hospice care behaviors among oncology nurses in order to improve the quality of life for terminally ill cancer patients.

**Supplementary Information:**

The online version contains supplementary material available at 10.1186/s12904-024-01385-8.

## Background

With an aging global population and a rising incidence of chronic disease, worldwide demand for hospice care (HC) is growing [1]. The global population of people aged 65 years or older is expected to reach 1.6 billion by 2050 [2], and there are now an estimated 18.19 million new cancer cases and 9.6 million cancer deaths per year worldwide [3]. In China specifically researchers estimate that by 2035, the proportion of people aged 60 and above will exceed 30% [4]. In addition, most cancer deaths in China occur between the ages of 60–74 [5]. On average, more than 11,100 people are diagnosed with new cancers and nearly 6,600 people die from cancer every day in China in recent years [6]. Although cancer incidence and deaths have been rapidly increasing, HC growth has not kept pace.

HC is a special type of care provided by members of a HC team, including physicians, nurses, home health aides, social workers, clergy or other counselors, and trained volunteers, who are dedicated to supporting the medical, psychological, and spiritual needs of terminally ill patients (with a life expectancy of six months or less) and their loved ones [7]. Currently, developed countries such as the United Kingdom and the United States have well-developed models of care, regulations, and educational systems regarding HC that support clinical standards for meeting the needs of patients and their families [8, 9]. In China, however, despite the implementation of numerous policies to foster the advancement of HC in recent years, there remains a significant deficit in HC expertise among health professionals [10–12]. Moreover, there is a scarcity of HC-related institutions, and their distribution is uneven, which leads to inadequate coverage [12, 13]. Additionally, due to the deep influence of Taoism, Confucianism, and Buddhism, most Chinese associate the word “death” with bad luck and refuse to discuss any topic even related to death [12]. Moreover, the traditional concept of life and death in China emphasizes the duration of life but neglects the quality, and most of China’s terminal patients still treat their diseases in order to prolong their lives during its final stages, when treatment is often most costly and has the lowest chance of success [10].

The development of HC in China has thus faced significant resistance due to deep-rooted traditional views of life and ethics, and the misconception that HC is equivalent to euthanasia [14]. Consequently, the quality of HC provided to patients in China is suboptimal [10, 13]. In an expert assessment of the quality of death in 81 countries in 2021, the quality of HC in mainland China was ranked 53rd, with a large gap compared to the world’s most advanced countries [15].

**Fig. 1:**
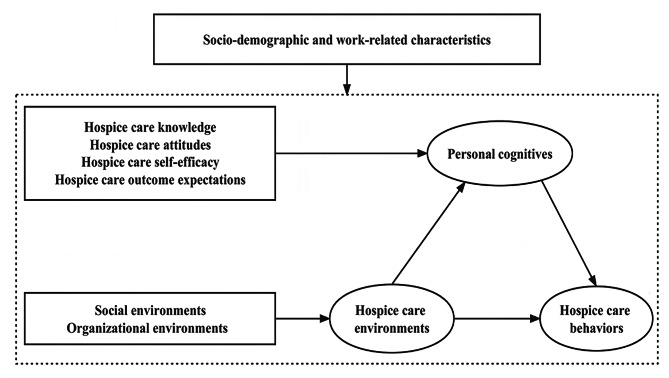
Conceptual framework

Oncology nurses’ HC behaviors are strongly associated with patients’ HC quality [16, 17]. However, there are no consistent findings regarding the factors associated with oncology nurses’ HC behaviors [18]. Currently, relatively few studies directly address nurses’ HC behaviors, and research on factors influencing this behavior in China has only focused on HC knowledge and HC attitudes [19, 20]. Environmental factors also influence individual behavior [21], but studies that explore the influence of environmental factors on HC behavior are relatively lacking. In addition, there are few systematic studies on HC behavior based on theoretical frameworks of any kind. Social Cognition Theory (SCT) is a commonly used behavioral research theory proposed by Bandura in the 1970s [22]. SCT holds that the individual, environment, and behavior have a mutually reinforcing dynamic relationship in which the individual and environment influence behavior. Cognitive factors are individual factors that should be focused on specifically, such as knowledge, attitudes, self-efficacy, and outcome expectations [23]. As a well-established theory, SCT has been widely used to explain and predict individual and group behavioral characteristics and to seek ways to change individual or group behavior [24–26]. Therefore, the SCT is useful for analyzing and interpreting research on nurses’ HC behaviors. The research framework we established based on SCT is shown in Fig. 1.

Since there is a lack of research on the HC behaviors and their influencing factors among oncology nurses in China, and because most of the HC behavior instruments in existing research are self-designed and lack systematic evaluation criteria and methods, the purpose of this study was to describe the current situation of HC behaviors of oncology nurses in China using a standardized scale and to explore the factors that influence these behaviors based on SCT in terms of demographic factors, personal cognitive factors, and environmental factors. This study not only provides a theoretical reference for promoting nurses’ HC participation but also lays the foundation for improving the quality of life of terminally ill patients.

## Methods

### Study design and aims

A cross-sectional study based on SCT was conducted to assess the current status of HC among oncology nurses and its influencing factors.

### Setting and sample

A convenience sampling method was used to select oncology nurses from six grade A tertiary hospitals in Hubei Province, China. We set out to use multiple linear regression, random forest regression analysis, and a BP neural network model for analysis. The multiple linear regression needed the sample size to be 10 to 20 times the number of independent variables [27]; however, the random forest regression analysis and BP neural network models currently have no clear criteria for sample size. The total number of independent variables in this study was 27, so the sample size needed to be between 270 and 540. Considering factors such as questionnaire rejection rate and invalid questionnaires, the target sample size was enlarged by 20%, to 324–648. The total sample size was actually achieved was 933 exceeding this target. The inclusion criteria were: (1) participating in nursing for at least one year, (2) working as an active registered nurse in an oncology unit, and (3) having no mental illness. The exclusion criteria were: (1) being a refresher or intern nurses and (2) not being currently engaged in clinical work. After excluding invalid questionnaires, 852 questionnaires were finally analyzed, with an effective response rate of 91.3%.

### Variables and measures

#### The general information questionnaire

Designed by our research team, the general information questionnaire contained: (1) sociodemographic information, including gender, age, marital status, ethnicity, religious beliefs, education, monthly income, and self-rated physical health status and (2) work-related information, including job title, position, type of hospital, work unit, years of work, job satisfaction, witness of the death of a terminally ill patient or relative, number of terminally ill patients cared for in the last year, HC education received as a student, number of HC training session attended after work, whether the work unit has an HC ward, willingness to engage in HC, frequency of sharing HC experiences with colleagues, and attainment of the HC nurse specialist certificate issued by the Chinese Nursing Association.

#### The hospice care behavior practices scale (HCBP)

The Hospice Care Behavior Practices Scale (HCBP) is used to evaluate the current status of HC behavior among healthcare professionals [28]. The tool has 14 items and uses a five-point Likert scale, with “never”, “rarely”, “sometimes”, “usually”, and “always” scoring from 1 to 5 points, respectively, and the higher the score, the higher the frequency of HC behavior. The scale has good reliability and validity, with a Cronbach’s α coefficient of 0.981 [29]. In this study, the Cronbach’s α was 0.943.

#### The hospice care knowledge scale (HCKS)

The Hospice Care Knowledge Scale (HCKS) is primarily used to assess the level of HC knowledge among healthcare workers [28]. This scale includes 15 entries divided into 5 dimensions: basic concepts and goals, pain and symptom management, psychology and spirituality, localized issues, and policy and organization. The scale consists of two types of questions: judgment questions (items 1–13) and single-choice questions (items 14–15), with one point for choosing the correct answer and a total score of 15 points. The higher the score, the higher the level of HC knowledge. The percentage of correct answers (%) = the number of people who answered the item correctly/the number of people who responded × 100%. Cronbach’s α coefficient for this scale was reported to be 0.686 by Jing’s research team [30], and the Cronbach’s α in this study was 0.711.

#### The hospice care attitude scale (HCAS)

The Hospice Care Attitude Scale (HCAS) is used to evaluate the status of HC attitudes among healthcare professionals [28] and consists of 25 items with 5 dimensions: perception of the threats from the worsening conditions of advanced patients, perception of the benefits of the quality of life promotion, perception of the benefits of better death preparation, perception of the barriers to providing palliative care, and subjective norms for the provision of HC. The scale is scored on a five-point Likert scale, with “totally disagree”, “partly disagree”, “neutral/nonsense”, “partly agree”, and “totally agree” being worth 1 through 5 points, sequentially but with dimension 1 and dimension 4 being scored in reverse. Higher scores on the scale indicate a more positive attitude toward HC. Jing’s team reported that the total Cronbach’s α coefficient of the scale was 0.868 [31], and the Cronbach’s α in this study was 0.877.

#### End-of-life professional caregiver survey scale (EPCS)

The End-of-Life Professional Caregiver Survey (EPCS) is a self-rating scale that can be used to measure the self-efficacy of HC for professionals across multiple disciplines [32–34]. The EPCS has good psychometric properties, with Lazenby et al. reporting Cronbach’s α values of 0.96, 0.95, 0.89, and 0.87 for the entire scale and each of its three dimensions, respectively [33]. Our research team revised and translated the EPCS to form a Chinese version that consists of 21 items in the three dimensions of “cultural, ethical, and national values (11 items)”, “patient-and family-centered communication (5 items)”, and “implementation of effective care (5 items)”. Our scale also uses a five-point Likert scale (from 0 to 4) to rate each entry, with higher total scores indicating higher levels of HC self-efficacy. The original scale has good reliability, with a total Cronbach’s α coefficient of 0.96 [35], and in the present study the Cronbach’s α of the Chinese version was 0.979.

#### The hospice care outcome expectancy scale (HCOES)

Currently, there is a lack of scales designed to assess HC outcome expectations for nurses. Our research team therefore developed the Hospice Care Outcome Expectations scale (HCOES) to assess nurses’ HC outcome expectations in strict accordance with the principles and processes of scale development recommended by DeVellis [36]. The HCOES consists of 7 items rated on a five-point Likert scale, with “completely disagree”, “disagree”, “neutral”, “agree”, and “completely agree” scored from 1 to 5, respectively. The total Cronbach’s α coefficient of the scale was 0.912. Furthermore, the content validity of HCOES was good, with the Item-Content Validity Index (I-CVI) ranging from 0.917 to 1.000, and a Scale-Content Validity Index (S-CVI) of 0.971. The construct validity of the scale was also good, with the 1-factor solution of the HCOES having a satisfactory model fit: χ^2^/df = 2.273, root-mean-square error of approximation (RMSEA) = 0.072, comparative fit index (CFI) = 0.990, incremental fit index (IFI) = 0.991, and goodness-of-fit index (GFI) = 0.972 [37]. Cronbach’s α was 0.925 for this study.

#### The hospice care environment scale (HCES)

As with expected outcomes, there is also a lack of scales specifically designed to assess the HC environment, so we developed the Hospice Care Environment Scale (HCES) along with the HCOES. The HCES consists of 13 items in 2 dimensions, including items 1–4 in the “social environment” dimension and items 5–13 in the “organizational environment” dimension. The five-point Likert scale was again used and in the same manner as in the HCOES. The total Cronbach’s α coefficient of the pre-assessment environment scale was 0.970, including 0.944 for the social environment dimension and 0.966 for the organizational environment dimension. Content validity for the HCES was good, with the I-CVI ranging from 0.917 to 1.000, and the S-CVI was 1. The construct validity of the scale was good as well: the 3-factor solution of the HCES had a satisfactory model fit: χ^2^/df = 2.689, RMSEA = 0.082, CFI = 0.982, IFI = 0.983, and GFI = 0.932 [37]. Cronbach’s α was 0.970 for this study.

### Data collection procedure

Data were collected from January to March, 2022. An electronic recruitment advertisement was created that stated the purpose and content of the study, the subjects to be recruited, the rights of the participants, and the contact details of the researchers. We created an online questionnaire using the web-based questionnaire platform “Questionnaire Star” and sent it to respondents who then read and completed the questionnaire independently. After data collection, a validity check was performed to screen out questionnaires with invalid responses.

### Ethical considerations

This study adhered to the guidelines of the Declaration of Helsinki. The Ethics Committee of Wuhan University School of Medicine issued an ethical approval for this study with the ethical number (2020YF2001). All participants were informed about the study and volunteered to participate in the study. In addition, the researchers obtained informed consent from all participants to indicate their consent before recruitment.

### Data analysis

Data were analyzed using SPSS 26.0 and R4.2.2 software. Descriptive statistics were used for participants’ demographic and work-related factors as well as the status of HC behaviors, HC knowledge, HC attitudes, HC self-efficacy, HC outcome expectations, and the HC environment. Potential behavior-influencing factors were first identified using independent-sample *t*-tests and one-way ANOVA, and Pearson correlation analysis was used to test the correlation between HC knowledge, HC attitudes, HC self-efficacy, HC outcome expectations, HC environment, and HC behaviors.

Three methods were used for multifactorial analysis: (1) Multiple linear regression. Tolerance (TOL) and the variance inflation factor (VIF) were used to determine the covariance among the independent variables, and no serious covariance was considered to exist among the independent variables if VIF < 5 and TOL > 0.2. Multiple linear regression analysis was performed to explore the factors influencing nurses’ HC behaviors, using nurses’ HCBP scores as the dependent variable and factors that were statistically significant (*P* < 0.05) in the univariate analysis as independent variables. A difference was considered statistically significant at *P* < 0.05. (2) Random forest regression model [38]. Random forest is a machine learning algorithm that can be used to build prediction models and screen variables. In particular, we used a deep learning random forest to construct a variable regression random forest that evaluated the mean square error of the model by out-of-bag error and screened the importance of the variables. To implement this, we utilized the mlr package in R, and screened the predictor variables by ridge regression, LASSO regression, and elastic net regression to determine the final characteristic variables. We then used a random regression forest to construct a model of predictor variables based on the dependent variable and to explore the accuracy of the model and the contribution of changes in each variable to changes in the dependent variable. (3) BP neural network [39]. The BP neural network model is a widely-used neural network algorithm at present. In this section, different neural network models were constructed using the neuralnet package in R, and then the optimal model was selected.

## Results

### General information and characteristics of participants

Among the 852 oncology nurses, 98.1% were female, and the average age of participants was 32.04 ± 6.55 years. Married nurses accounted for the majority (72.2%), and the vast majority of the nurses reported no religious beliefs (95.2%). The majority (76.5%) had a bachelor’s degree or above, and 61.3% of the nurses rated themselves as having good or very good health status (Table 1). For work-related characteristics, 60.8% of the participants had junior titles. The average number of working years was 9.98 ± 7.21. Only 8.7% of nurses often or always shared HC experiences with colleagues (See Supplementary Table 1, Additional File 1).

**Table 1:** Demographic characteristics of participants (*N* = 852)

Variable	Category	*N* (%)	HC behavior ($\stackrel{-}{x}$±s)	*t*/*F*	*P*
Gender	Male	16 (1.9)	51.75 ± 10.82	0.261	0.794
	Female	836 (98.1)	51.12 ± 9.50		
Age	≤ 25	136 (16.0)	50.03 ± 9.72	2.073	0.083
	26–30	240 (28.2)	51.40 ± 9.48		
	31–35	270 (31.7)	50.38 ± 9.24		
	36–40	112 (13.1)	52.66 ± 9.35		
	≥ 41	94 (11.0)	52.40 ± 10.12		
Marital status	Others	237 (27.8)	50.92 ± 10.12	-0.399	0.690
	Married	615 (72.2)	51.22 ± 9.29		
Ethnicity	Han	830 (97.4)	51.04 ± 9.47	-1.750	0.080
	Minority	22 (2.6)	54.64 ± 11.08		
Religious beliefs	No	811 (95.2)	51.21 ± 9.45	1.05	0.294
	Yes	41 (4.8)	49.61 ± 10.85		
Education	Junior college and below	200 (23.5)	50.19 ± 10.03	-1.604	0.109
	Bachelor’s degree or above	652 (76.5)	51.42 ± 9.35		
Monthly Income	<5000	414 (48.6)	50.06 ± 9.63	-3.231	0.001
	≥ 5000	438 (51.4)	52.15 ± 9.31		
Self-rated physical health status	Very good	175 (20.5)	52.91 ± 9.84	4.562	0.004
	Good	348 (40.8)	51.47 ± 9.43		
	General	298 (35.0)	49.99 ± 9.22		
	Bad	31 (3.6)	48.32 ± 10.06		

### Current status of HC behaviors

The mean score of behaviors was 50.47 ± 10.56, with a mean item score of 3.61 ± 0.75. The three highest scoring behaviors were “pain assessment of patients (4.21 ± 0.91)”, “satisfying the physical and mental needs of dying patients (4.04 ± 0.92)”, and “creating good relationships between the medical staff and family members (4.02 ± 0.87)”. The two lowest-scoring behaviors were “proactively recommending medical institutions for hospice care to terminally ill patients and their families (2.55 ± 1.10)” and “proactively talking to patients and families about death-related topics for patients who are critically ill and cannot be reversed (2.87 ± 1.03) (Table 2).

**Table 2:** HC behavior scores (*N* = 852)

Items	Total score ($\stackrel{-}{x}$±s)	Often/Always	
		*N*	Percentage (%)
Total HC behavior score	50.47 ± 10.56		
1. You proactively talk to patients and families about death-related topics for patients who are critically ill and cannot be reversed.	2.87 ± 1.03	191	21.98
2. Proactively recommend medical institutions for HC to terminally ill patients and their families	2.55 ± 1.10	159	18.30
3. Talk to the patient’s family proactively about “respecting the patient’s wishes”	3.25 ± 1.08	359	41.31
4. Alleviating pain and discomfort in terminally ill patients (pain management)	3.87 ± 0.99	587	67.55
5. Make pain assessment of patients.	4.21 ± 0.91	709	81.59
6. Reduce unnecessary treatment costs	3.87 ± 0.97	596	68.59
7. Satisfy the physical and mental needs of dying patients	4.04 ± 0.92	667	76.76
8. Explain the expected dying process to the patient and family	3.38 ± 1.10	394	45.34
9. Tell families specific things they can do to provide meaningful services to patients	3.75 ± 0.95	545	62.72
10. Understand the wishes and pain of family to help them.	3.88 ± 0.89	605	69.62
11. Create good relationship between the medical staff and family members.	4.02 ± 0.87	665	76.53
12. Coordinate the media resources of medical, social, psychological and spiritual care	3.64 ± 1.04	503	57.88
13. Help risk grieving families to get through better.	3.64 ± 0.99	500	57.54
14. Guide the management of afterwards and funeral preparation for families	3.47 ± 1.07	428	49.25

### Current status of other key variables

The mean score of HC knowledge of respondents was 8.85 ± 2.97, ranging from 0 to 15 (See Supplementary Table 2, Additional File 1). Specifically, the HC attitude score was 93.73 ± 13.60, with a score range of 42–125 and a mean item score of 3.75 ± 0.54 (See Supplementary Table 3, Additional File 1); the HC self-efficacy score was 52.62 ± 19.06, with a score range of 0–84 and a mean item score of 2.51 ± 0.91 (See Supplementary Table 4, Additional File 1); the HC outcome expectancy score was 24.55 ± 5.11, with a score range of 7–35 and an item score of 3.51 ± 0.73 (See Supplementary Table 5, Additional File 1); and the HC environment score was 45.43 ± 10.13, with a score range of 13–65 and a mean item score of 3.49 ± 0.78 (See Supplementary Table 6, Additional File 1).

### Correlation between key variables

The results of Pearson correlation analysis showed that HC knowledge, HC attitude, HC self-efficacy, HC outcome expectancy, and HC environment were each significantly and positively correlated with HC behavior (*p* < 0.01) (Table 3).

**Table 3:** Pearson correlation coefficients between key study variables (*N* = 852)

Entries	1	2	3	4	5	6
1. HC knowledge	1					
2. HC attitude	0.210**	1				
3. HC self-efficacy	0.199**	0.354**	1			
4. HC outcome expectancy	0.175**	0.310**	0.625**	1		
5. HC environment	0.202**	0.289**	0.646**	0.689**	1	
6. HC behavior	0.196**	0.391**	0.508**	0.378**	0.456**	1

Note: **P < 0.01

### Multifactor analysis of HC behaviors

#### Multiple linear regression

Prior to our regression analyses, we checked the linearity, multivariate normality, and homoskedasticity of the variables. The results of the tests for multicollinearity using TOL and VIF values revealed no multicollinearity problems for any variables (TOL>0.2; VIF: 1.039–2.422). Based on the results of the univariate analysis, five variables were used in our multiple linear regression equation: position, frequency of sharing HC experiences with colleagues, HC attitude, HC self-efficacy, and HC environment. The equation had an R^2^ of 0.426 and an adjusted R^2^ of 0.413, indicating that the five variables described above explained 41.3% of the variance in HC behavior. In descending order of contribution this was: HC self-efficacy, frequency of sharing HC experiences with colleagues, HC environment, HC attitude, and position (Table 4).

**Table 4:** Multiple linear regression results for HC-behavior-influencing factors

Variables	B	SE	β	*t*	*P*	VIF
Constant	16.093	2.581		6.235	<0.001	
HC self-efficacy	0.136	0.019	0.268	7.143	<0.001	2.042
Frequency of sharing HC experiences with colleagues	2.755	0.386	0.236	7.144	<0.001	1.584
HC environment	0.186	0.039	0.196	4.790	<0.001	2.422
HC attitude	0.113	0.021	0.161	5.423	<0.001	1.286
Position	2.319	1.026	0.073	2.261	0.024	1.497

Note: R^2^ = 0.426,adjusted R^2^ = 0.413;F = 34.319, *P*<0.001

#### Random forest regression

An elastic net model, LASSO regression model, linear regression model, and ridge regression model were first constructed, then their parameters were encapsulated using the makeTuneWrapper() function, then the hyperparameters were adjusted using the benchmark() function. The mean squared error of each model as well as the final screened variables were then compared using triple-folded cross-validation repeated 10 times. This comparison showed that the LASSO regression model had the smallest mean square error at 61.737.

**Fig. 2:**
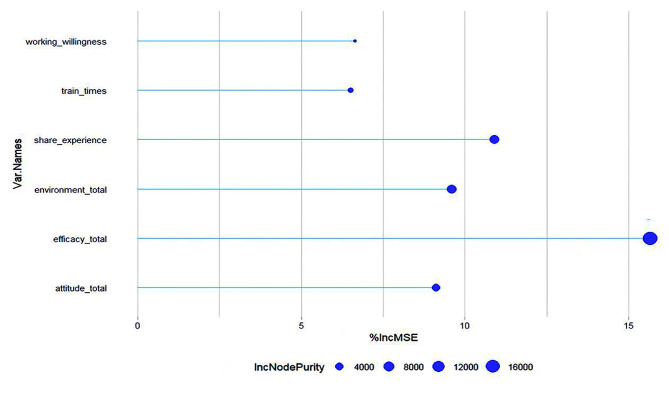
The importance of each variable in the random forest model

Based on the feature variables screened by the LASSO regression, the random forest model was constructed using the regr.randomForest() function and the randomForest software package. When the number of random forest trees (ntree) was 53, the number of randomly selected samples per tree (mtry) was 943, the minimum number of samples allowed for leaf nodes (nodesize) was 2, and the maximum number of nodes in each tree (maxnodes) was 18, the minimum mean squared residuals of the model were obtained as 56.62. The order of importance between the variables in this model is shown in Fig. 2. In the order most to least important this was HC self-efficacy, frequency of sharing HC experiences with colleagues, HC environment, HC attitudes, willingness to engage in HC, and number of HC training sessions attended after work.

#### BP neural network

**Fig. 3:**
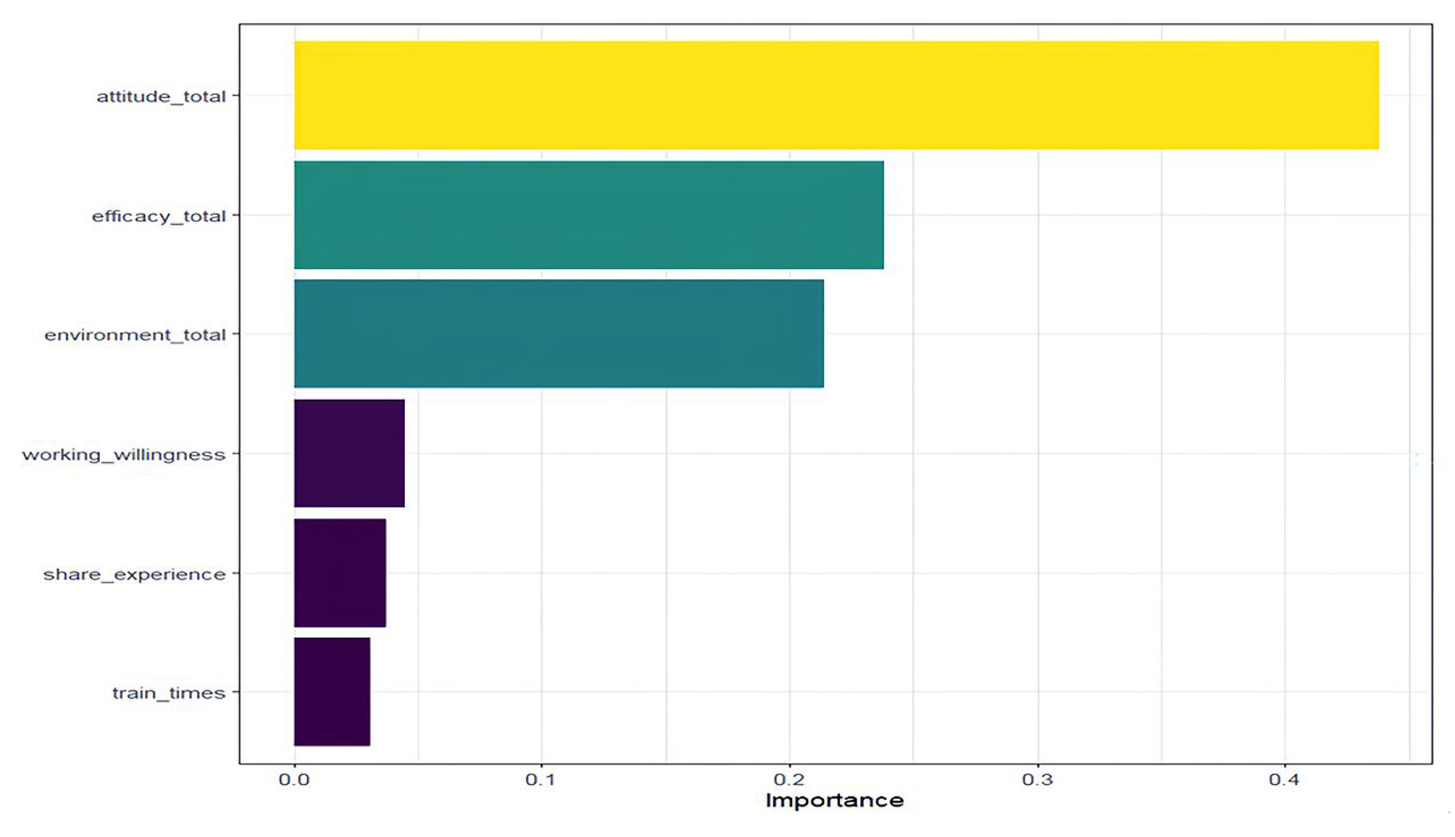
The importance of each variable in the BP neural network model

The dataset of feature variables selected by random forest regression was randomly divided into a training set and a test set, where the training set accounted for 70% of the data, and the test set accounted for 30%. The logistic activation function and the tran hyperbolic activation function were each used to construct both a simple and a complex BP neural network model. Comparing the sum of squared errors of the above four models, the complex BP neural model constructed by the logistic activation function had the smallest sum of squared errors in both the training set and the test set, which were 17.25 and 13.35, respectively. In this model the independent variables, in descending order of influence, were HC attitudes, HC self-efficacy, HC environment, willingness to engage in HC, frequency of sharing HC experiences with colleagues, and number of HC training sessions attended after work (Fig. 3).

## Discussion

In this study, we assessed the HC behaviors of 852 Chinese oncology nurses using HCBP and explored the factors associated with these behaviors based on SCT in terms of demographic, personal cognitive, and environmental factors. The results show that the mean score of HC for Chinese oncology nurses was 50.47 ± 10.56. Multiple linear regression, random forest regression, and BP neural network models all showed that the frequency of sharing HC experience with colleagues, HC attitude, HC self-efficacy, and HC environment were positively associated with HC behavior.

Our study revealed that oncology nurses practiced HC at a moderate to high frequency, which is generally consistent with recent findings [29, 40], but higher than earlier studies [41, 42]. The behavior of “making pain assessment of patients” was the most common, followed by “satisfying the physical and mental needs of dying patients” and “creating good relationships between the medical staff and family members”, which is consistent with the findings of Xu et al. [29], indicating that oncology nurses tend to focus on the physical and psychological care of patients and the care of their families. However, only about 20% of the nurses frequently or always engaged in the behaviors of “proactively recommending medical institutions for HC to terminally ill patients and their families” and “proactively talking to patients and families about death-related topics for terminally ill patients”.

Currently, there are not many hospitals or other institutions that offer HC in China, and a hierarchical diagnosis and treatment system for HC has yet to be formed. Although physicians are responsible for patient referral and discharge decisions, nurses are often faced with questions from patients and family members about where they should receive follow-up HC after discharge. In our study, nurses rarely proactively provided such information, which may be related to their lack of knowledge about HC institutions, their busy work schedules, or the perception that providing such advice is the primary responsibility of physicians. If nurses can improve their knowledge related to this behavior, such as which institutions can provide HC and whether these institutions are suitable for the patient in terms of price, location, and other factors, and commit to participating in it, this may help promote the spread of HC. In regard to proactively talking to patients and families about death-related topics, Mei et al. [40] also found that clinical nurses avoided talking about death with patients. At present, there is a lack of life-and-death education in China, so the acceptance of HC by the general public is poor. Therefore, we recommend that China accelerate the development of a hierarchical diagnosis and treatment system for HC, and adopt a combination of online and offline methods to carry out life and death education for the whole population together with HC education for healthcare professions.

In terms of demographic and work-related factors, the results of all three of our multifactor analyses showed that the frequency of sharing HC experiences with colleagues was a predictor of nurses’ HC delivery, which is consistent with the study by Wu et al. [18]. The more frequently nurses shared their HC experiences with colleagues, the more frequently they performed HC. We therefore suggest that measures can be taken to encourage nursing staff to share more HC experiences in order to promote more and better HC. In addition, although position only appeared and had the least effect on HC behaviors in multiple linear regressions, it gives us guidance on the direction of intervention. Nurse managers and directors of nursing had more HC behaviors compared to general nurses, which may be related to the fact that they have more time to provide psychological care, spiritual care, and deeper communication with patients. It may also be related to the fact that they tend to be more professional, better at communicating, and have learned more about HC. Thus, in the future, we propose to increase the number of nurses so that all nurses will have more time to carry out high-quality HC. It is also important to strengthen the training of nurses in communication skills, especially in psychological and spiritual matters.

Furthermore, the willingness to engage in HC and the number of times a nurse attended HC training sessions after work were not statistically significant in the multiple linear regression, but these two were predictors of HC in both the random forest and BP neural network, which is an indication of a direction for future research. Multiple linear regression modeling is widely used, but it requires a linear relationship between the independent and dependent variables. However, many variables in our study were categorical variables. Random forest regression, can take into account the interaction effects and nonlinear relationships ignored by multiple linear regression, and it is also robust to outliers. Hence, it is better in many ways than other machine-learning algorithms. Moreover, the BP neural network model can also deal with nonlinear relationships that may be ignored by multiple linear regression; it can handle high correlation between variables and can deal with the problem of possible covariance between the influencing factors. Additionally, there are no requirements on data distributions (whether or not it is normally distributed). Therefore, the focus should be on the factors that were recognized by all three models. Important factors that are not all recognized by the three models should be investigated and validated in future studies.

Regarding individual cognitive factors, we found that although HC knowledge, HC attitudes, HC self-efficacy, and HC outcome expectations were positively associated with HC behavior in univariate analysis, the results of the multifactor analysis all showed that only HC attitudes and HC self-efficacy were significant predictors of HC behavior. Huijer [43] and Xu et al. [44] also showed that HC attitudes were an independent influence on HC behavior and that the more positive the nurses’ attitudes toward HC, the more willing they were to work in HC and the better their implementation of HC behaviors. Although there is a lack of research on HC self-efficacy and HC behavior, studies have shown that nurses with higher self-efficacy are more engaged in nursing [45]. Knowledge tends to influence one’s attitudes and behaviors, but our results showed that knowledge was not a predictor of HC behavior, similar to the findings of Gilissen et al. [46]. Gilissen’s study found that nurses’ knowledge was not related to the implementation of a pre-established health care plan but that their self-efficacy was related to it.

According to SCT, outcome expectancy is one of the more important personal cognitive factors that influences behaviors, and this study showed that HC outcome expectancy was associated with HC behavior, although multifactor analysis showed that it was not a predictor of HC behavior. One qualitative study showed that nurses have many gains or growth opportunities related to HC that benefit themselves, but whether these gains become motivators or facilitators for nurses to engage in HC behaviors needs further study [47–49]. No previous studies have specifically investigated and analyzed nurses’ HC outcome expectations and their impact on HC behaviors either, and more research should be conducted in the future to clarify the relationship between the two. Therefore, we suggest that measures to promote nurses’ HC should focus on improving nurses’ HC attitudes and HC self-efficacy rather than just improving nurses’ HC knowledge.

HC environment was also shown to be an important predictor of HC behavior by all multifactor analyses. Little previous research has been conducted specifically on the HC environment and its effect on HC behavior, but one recent study showed that the nursing work environment was an independent predictor of HC and the level of support for HC work by nurse managers was an independent predictor of HC behavior [18], which supports the results of our study to some extent. According to SCT, the individual, the environment, and behavior are in a mutually reinforcing, dynamic relationship. These elements interact with each other, and sometimes the environmental factors can have a strong constraining effect on behavior. HC in China started late compared to its demographic needs. Although in recent years the state has issued several policies and documents aimed at promoting the development of HC, China’s HC is still in an exploratory stage, and environmental factors are important constraints on the development of HC in some regions. In Hubei province, for example, where this study was conducted, there is not enough awareness of HC among the general public and medical and nursing professionals compared to areas such as Shanghai and Beijing in China. We therefore reiterate our suggestion that the Chinese government take active measures to improve the awareness of HC among the general public and medical and nursing professionals in particular, and to improve the environment in which HC is practiced.

### Limitations

Our results should be considered in light of several limitations. Firstly, this was a cross-sectional study that only dealt in correlation rather than causation between HC behaviors and influencing factors. We therefore suggest that a longitudinal study be conducted in the future to explore the causality of personal and environmental factors on HC behaviors. Secondly, the convenience sampling method used in this study may have resulted in selection bias, thus limiting extrapolation of the results. Future studies should be conducted in more districts and use more rigorous sampling methods in order to improve the reliability and generalizability of the results. Finally, this study did not address the psychological condition of nurses when exploring the factors that affect their HC behaviors. Future studies should therefore examine psychological condition factors in order to provide a more comprehensive analysis of nurses’ HC behaviors.

## Conclusion

This study analyzed the current situation and factors influencing HC among oncology nurses in six grade A tertiary hospitals in Hubei Province, China. The results suggest that the frequency of HC behaviors among oncology nurses is moderately good but that measures should be taken to promote HC among nurses even further. The results our multifactor analysis suggest that personal cognitive (HC knowledge, HC attitude, HC self-efficacy, and HC outcome expectancy) and environmental factors may be key intervention directions in which to promote nurse HC behaviors. This study not only provides a theoretical reference for promoting HC behaviors in nurses but also lays the foundation for improving the quality of life of terminally ill patients.

### Electronic supplementary material

Below is the link to the electronic supplementary material.

Supplementary Material 1

## Data Availability

All data generated or analyzed during this study will be made available upon request from the corresponding author.

## Abbreviations

HC: Hospice care
SCT: Social Cognition Theory
HCBP: Hospice Care Behavior Practices Scale
HCKS: Hospice Care Knowledge Scale
HCAS: Hospice Care Attitude Scale
EPCS: End-of-Life Professional Caregiver Survey Scale
HCOES: Hospice Care Outcome Expectancy Scale
I-CVI: Item-Content Validity Index
S-CVI: Scale-Content Validity Index
RMSEA: Root-mean-square error of approximation
CFI: Comparative fit index
IFI: Incremental fit index
GFI: Goodness-of-fit index
HCES: Hospice Care Environment Scale
TOL: Tolerance
VIF: Variance Inflation Factor
