# Supplementary material for: Behaviors and influencing factors of Chinese oncology nurses towards hospice care: a cross-sectional study based on social cognitive theory in 2022
Source: BMC Palliat Care. 2024 Feb 23;23:53. doi: 10.1186/s12904-024-01385-8 (PMC10885468; doi:10.1186/s12904-024-01385-8)
Supplement: Supplementary file 1 — Supplementary Material 1 [file 12904_2024_1385_MOESM1_ESM.docx]

**Supplementary Table 1** Work-related information of participants (N = 852).

| Variable | Category | N (%) | Hospice care behavior ($\bar{x}$±s) | t/F | P |
| --- | --- | --- | --- | --- | --- |
| Job title | Primary | 518 (60.8) | 50.63±9.54 | 4.820 | 0.008 |
|  | Intermediate | 292 (34.3) | 51.44±9.45 |  |  |
|  | Associate senior and above | 42 (4.9) | 55.24±8.91 |  |  |
| Position | General nurse | 768 (90.1) | 50.78±9.49 | -3.262 | 0.001 |
|  | Head nurse or director of nursing | 84 (9.9) | 54.33±9.28 |  |  |
| Type of hospital | General hospital | 733 (86.0) | 51.18±9.50 | 0.321 | 0.748 |
|  | Oncology specialized hospital | 119 (14.0) | 50.87±9.71 |  |  |
| Work unit | Medical oncology | 380 (44.6) | 51.94±9.22 | 2.551 | 0.079 |
|  | Surgical oncology | 138 (16.2) | 50.80±9.19 |  |  |
|  | Other oncology departments | 334 (39.2) | 50.36±9.94 |  |  |
| Years of work | 1-5 | 238 (27.9) | 50.17±9.56 | 2.447 | 0.045 |
|  | 5-10 | 292 (34.3) | 50.89±9.34 |  |  |
|  | 10-15 | 181 (21.2) | 51.33±9.42 |  |  |
|  | 15-20 | 61 (7.2) | 54.15±9.58 |  |  |
|  | ＞20 | 80 (9.4) | 52.15±9.91 |  |  |
| Job satisfaction | Very dissatisfied | 41 (4.8) | 53.49±11.30 | 8.331 | ＜0.001 |
|  | Discontented | 48 (5.6) | 47.25±9.54 |  |  |
|  | General | 338 (39.7) | 50.01±9.28 |  |  |
|  | Satisfied | 355 (41.7) | 51.54±9.24 |  |  |
|  | Very satisfied | 70 (8.2) | 55.74±9.04 |  |  |
| Witness of the death of a terminally ill patient or relative | No | 83 (9.7) | 49.20±10.64 | -1.946 | 0.052 |
|  | Yes | 769 (90.3) | 51.34±9.38 |  |  |
| Number of terminally ill patients cared for in the last year | 0 | 191 (22.4) | 48.46±9.76 | 8.575 | ＜0.001 |
|  | 1-10 | 482 (56.6) | 51.38±9.39 |  |  |
|  | 11-30 | 115 (13.5) | 53.50±8.97 |  |  |
|  | 31or above | 64 (7.5) | 53.02±9.12 |  |  |
| HC education received as a student | None | 314 (36.9) | 49.53±10.02 | 11.981 | ＜0.001 |
|  | Rarely | 281 (33.0) | 51.13±9.00 |  |  |
|  | Sometimes | 228 (26.8) | 52.29±8.79 |  |  |
|  | Quite a lot | 29 (3.4) | 59.55±9.16 |  |  |
| Number of HC training session attended after work | 0 | 297 (34.9) | 47.28±9.43 | 32.028 | ＜0.001 |
|  | 1-3 | 402 (47.2) | 52.37±8.66 |  |  |
|  | 4-6 | 88 (10.3) | 54.75±9.55 |  |  |
|  | ≥7 | 65 (7.6) | 56.22±8.84 |  |  |
| Work unit has an HC ward | No | 652 (76.5) | 50.13±9.28 | -5.653 | ＜0.001 |
|  | Yes | 200 (23.5) | 54.41±9.59 |  |  |
| Willingness to engage in HC | No | 307 (36.0) | 47.39±9.08 | -9.007 | ＜0.001 |
|  | Yes | 545 (64.0) | 53.24±9.12 |  |  |
| Frequency of sharing HC experiences with colleagues | None | 116 (13.6) | 44.84±10.00 | 61.568 | ＜0.001 |
|  | Rarely | 418 (49.1) | 49.25±8.54 |  |  |
|  | Sometimes | 244 (28.6) | 54.76±7.82 |  |  |
|  | Often | 74 (8.7) | 59.66±8.85 |  |  |
| Obtain the HC nurse specialist certificate issued by the Chinese Nursing Association | No | 835 (98.0) | 51.04±9.50 | -2.081 | 0.038 |
|  | Yes | 17 (2.0) | 55.88±9.50 |  |  |

**Supplementary Table 2** HC knowledge scores of oncology nurses (n = 852).

| Items | Correct option | Correct number of people | Correct rate (%) |
| --- | --- | --- | --- |
| 1. The provision of hospice care requires emotional detachment. | false | 172 | 20.2 |
| 2. The highest priority of the hospice care team is to give appropriate counseling and management of psychological, social and spiritual problems of patients and families. | true | 739 | 86.7 |
| 3. The World Health Organization's standardized medication for cancer pain is based on a 3-tiered analgesic ladder of mild, moderate and severe pain. | true | 765 | 89.8 |
| 4. The hospice care team provides bereavement care (grief counseling support) to families after a patient's death. | true | 664 | 77.9 |
| 5. Hospice care at home is in line with our folk customs. | true | 514 | 60.3 |
| 6. For bereavement care, children may be allowed to attend the funeral and even participate in the preparations. | true | 535 | 62.8 |
| 7. In the advanced stages of the disease, drugs that can cause respiratory depression are appropriate for the treatment of severe respiratory distress. | true | 357 | 41.9 |
| 8. Applying mirabilite at Shenque point can relieve ascites. | true | 373 | 43.8 |
| 9. Fatigue or anxiety can lower the pain threshold. | true | 342 | 40.1 |
| 10. Men are usually quicker to reconcile their grief than women. | false | 260 | 30.5 |
| 11. Individuals taking morphine should also follow enteral therapy. | true | 467 | 54.8 |
| 12. The "Health China 2030" plan outline includes a proposal to strengthen the construction of hospice care medical institutions. | true | 637 | 74.8 |
| 13. In the terminal phase, morphine acupoint injections can be used to relieve cancer pain. | true | 572 | 67.1 |
| 14. The most authoritative health care planning guidelines recommend that hospice care is most appropriate for： a. A dedicated multidisciplinary hospice team, including the family's general practitioner, b. General Practitioners, c. Multi-specialty team led by pain management specialists, d. Cooperation between specialist nurses and anesthesiologists, e. Specialist nurses | a | 730 | 85.7 |
| 15. End-stage music therapy for advanced malignancy aims to improve the quality of life or the ability to perform activities of daily living, with effects that do not include: a. Relief of physical pain, b. Entertainment and friendship, c. Expressing emotions, d. Inducing memories of the past, e. Comfort for grief | b | 409 | 48.0 |

**Supplementary Table 3** HC attitude scores of oncology nurses (n = 852).

| Items | Total score ($\bar{x}$*±s*) |
| --- | --- |
| Total Score | 93.73±13.60 |
| 1. Perception of the threats from the worsening conditions of advanced patients is: | 15.90±4.63 |
| 1.1 Uncomfortable to take care of advanced cancer patients. | 3.66±1.21 |
| 1.2 Hopeless for the cure. | 3.12±1.26 |
| 1.3 Unable to easily face dying process and distress. | 3.17±1.21 |
| 1.4 Makes me feel weakness. | 3.08±1.23 |
| 1.5 I feel guilty when amine patient dies. | 2.86±1.24 |
| 2. Perception of the benefits for the life quality promotion is: | 21.43±4.49 |
| 2.1 Able to promote life quality and keep the dignity. | 4.29±0.95 |
| 2.2 Able to die peacefully and have a good death. | 4.31±0.93 |
| 2.3 Having care and accompanied by medical team. | 4.31±0.92 |
| 2.4 Emotional support. | 4.29±0.93 |
| 2.5 Able to have family support. | 4.23±0.93 |
| 3. Perception of the benefits for better death preparation is: | 20.62±3.89 |
| 3.1 Respect for patient's religion and burial rites. | 4.34±0.85 |
| 3.2 Help to die at home. | 3.82±1.03 |
| 3.3 Better communication with advanced patients. | 4.25±0.85 |
| 3.4 Help medical staff to take care of patients better. | 4.29±0.86 |
| 3.5 Avoid the idea of euthanasia. | 3.92±1.04 |
| 4. Perception of the barriers to provide palliative care is: | 19.67±5.72 |
| 4.1 Shorten patient's life, just like euthanasia. | 3.55±1.29 |
| 4.2 No active treatment for physical symptoms. | 3.44±1.30 |
| 4.3 Make patients feel hopeless. | 3.82±1.22 |
| 4.4 Advanced patients have many difficult symptoms. | 2.65±1.20 |
| 4.5 Keep providing long-term hospice care service will lose enthusiasm. | 3.19±1.22 |
| 4.6 Patient and/or family refusal to accept hospice philosophy and services | 3.02±1.14 |
| 5. Subjective norms for provision of hospice care: | 16.11±3.47 |
| 5.1 It is meaningful. | 4.26±0.91 |
| 5.2 I experienced the death of my family member, which affected me to provide hospice care. | 3.80±1.08 |
| 5.3 It is a part of duty on medical staff. | 4.09±0.95 |
| 5.4 With the approval and support of department leader, colleagues, relatives and friends, I was encouraged to provide hospice care. | 3.96±0.99 |

**Supplementary Table 4** HC self-efficacy scores of oncology nurses (n = 852).

| Items | Total score ($\bar{x}$*±s*) |
| --- | --- |
| Total score | 52.62±19.06 |
| 1. Cultural, Ethical and National Values: | 26.69±10.76 |
| 1.1 I am comfortable addressing ethical issues related to end-of-life care/hospice care/palliative care. | 2.56±1.07 |
| 1.2 I can work with patients and their families on spiritual issues (such as the meaning and value of life). | 2.54±1.09 |
| 1.3 I am comfortable with the religious beliefs and cultural aspirations of patients and families. | 2.56±1.09 |
| 1.4 I can comfortably provide grief counseling to patients' families. | 2.51±1.08 |
| 1.5 I am familiar with the cultural factors that influence end-of-life care. | 2.45±1.11 |
| 1.6 I can tell when a patient is appropriate for transfer to a hospice facility. | 2.37±1.12 |
| 1.7 I am familiar with the principles of hospice care and national guidelines. | 2.31±1.17 |
| 1.8 I can effectively help patients maintain continuity of care when they change care locations. | 2.44±1.11 |
| 1.9 I am confident that I can handle a patient's request for assistance with suicide. | 2.32±1.15 |
| 1.10 I have personal resources to help me meet my own needs when caring for terminally ill patients and families. | 2.20±1.21 |
| 1.11 I feel that my workplace provides support resources for staff caring for terminally ill patients. | 2.43±1.09 |
| 2. Patient- and family-centered communication: | 13.22±4.70 |
| 2.1 I can comfortably help the patient's family accept the patient's poor prognosis. | 2.58±1.04 |
| 2.2 I can work with patients and families to set goals for their care. | 2.74±0.98 |
| 2.3 I am comfortable discussing with patients and families their personal choices and self-determination. | 2.62±1.05 |
| 2.4 I can comfortably organize and participate in discussions about whether to do CPR | 2.63 ±1.09 |
| 2.5 I can assist the patient's family and other relatives and friends through the grieving period after the death of the patient. | 2.65±1.02 |
| 3. Implementation of effective care: | 12.70±4.79 |
| 3.1 I can recognize near death (signs and symptoms). | 2.73±1.00 |
| 3.2 I know how to use non-pharmacological therapies to manage my patients' symptoms. | 2.41±1.08 |
| 3.3 I can allay the fears of patients and family members about addiction to painkillers. | 2.46±1.11 |
| 3.4 I encourage patients and families to develop pre-established medical care plans. | 2.58±1.03 |
| 3.5 I can be by the side of the dying. | 2.52±1.08 |

**Supplementary Table 5** HC outcome expectancy scores for oncology nurses (n = 852).

| Items | Total score ($\bar{x}$*±s*) |
| --- | --- |
| Total score | 24.55±5.11 |
| 1. Providing hospice care has made me more content within myself. | 3.50±0.90 |
| 2. Providing hospice care has allowed me to receive better wages and benefits. | 3.05±0.95 |
| 3. Providing hospice services has helped me and my colleagues to help each other and to have a better relationship. | 3.60±0.86 |
| 4. Providing hospice services has had a better impact on my family. | 3.44±0.90 |
| 5. Providing hospice care has made me more self-affirming and fulfilling. | 3.62±0.86 |
| 6. Providing hospice care has allowed me to have better personal growth and development. | 3.67±0.83 |
| 7. Providing hospice care has helped me gain more recognition and respect. | 3.67±0.84 |

**Supplementary Table 6** HC environment scores for oncology nurses (n = 852).

| Items | Total score ($\bar{x}$*±s*) |
| --- | --- |
| Total score | 45.43±10.13 |
| 1. Social Environment: | 14.03±3.37 |
| 1.1 The government administration has a well-established hospice care policy. | 3.51±0.90 |
| 1.2 The community and residents are aware of and recognize hospice care services. | 3.42±0.94 |
| 1.3 End-stage patients recognize and accept hospice care services. | 3.54±0.89 |
| 1.4 Families of end-stage patients recognize and accept hospice care services. | 3.57±0.87 |
| 2. Organizational Environment: | 31.41±7.35 |
| 2.1 My department has a hospice care management system. | 3.44±0.93 |
| 2.2 My department has incentives for hospice care work. | 3.41±0.94 |
| 2.3 The managers in my department recognize and actively promote hospice care. | 3.56±0.89 |
| 2.4 The physicians in my department recognize and actively work on hospice care. | 3.52±0.89 |
| 2.5 The nurses on my unit recognize and actively practice hospice care. | 3.60±0.86 |
| 2.6 The medical and nursing staff in my department are able to work actively together to accomplish hospice care. | 3.62±0.84 |
| 2.7 Our unit has adequate medical staff to provide hospice care. | 3.43±0.93 |
| 2.8 Our department integrates multidisciplinary staff (e.g., dieticians, social workers, volunteers, etc.) to provide hospice care to patients. | 3.46±0.94 |
| 2.9 Our department has good environmental facilities (e.g. single room, double room, aromatherapy oil, music, etc.) to provide hospice care for patients. | 3.37±1.00 |
